# ETV5 links the FGFR3 and Hippo signalling pathways in bladder cancer

**DOI:** 10.1038/s41598-018-36456-3

**Published:** 2019-04-05

**Authors:** Erica di Martino, Olivia Alder, Carolyn D. Hurst, Margaret A. Knowles

**Affiliations:** University of Leeds, Leeds Institute of Medical Research at St James’s, St. James’s University Hospital, Leeds, LS9 7TF UK

## Abstract

Activating mutations of fibroblast growth factor receptor 3 (FGFR3) are common in urothelial carcinoma of the bladder (UC). Silencing or inhibition of mutant FGFR3 in bladder cancer cell lines is associated with decreased malignant potential, confirming its important driver role in UC. However, understanding of how FGFR3 activation drives urothelial malignant transformation remains limited. We have previously shown that mutant FGFR3 alters the cell-cell and cell-matrix adhesion properties of urothelial cells, resulting in loss of contact-inhibition of proliferation. In this study, we investigate a transcription factor of the ETS-family, ETV5, as a putative effector of FGFR3 signalling in bladder cancer. We show that FGFR3 signalling induces a MAPK/ERK-mediated increase in ETV5 levels, and that this results in increased level of TAZ, a co-transcriptional regulator downstream of the Hippo signalling pathway involved in cell-contact inhibition. We also demonstrate that ETV5 is a key downstream mediator of the oncogenic effects of mutant FGFR3, as its knockdown in FGFR3-mutant bladder cancer cell lines is associated with reduced proliferation and anchorage-independent growth. Overall this study advances our understanding of the molecular alterations occurring during urothelial malignant transformation and indicates TAZ as a possible therapeutic target in FGFR3-dependent bladder tumours.

## Introduction

Fibroblast growth factors (FGF) and their four tyrosine kinase receptors (FGFR1-4) activate multiple downstream cellular signalling pathways, such as MAPK/ERK, PLCγ1, PI3K and STATs, and regulate a variety of physiological processes, encompassing embryogenesis, angiogenesis, metabolism, and wound healing^1–3^. Dysregulation of FGF signalling is found in many pathological conditions, including craniofacial and skeletal dysplasia syndromes, other developmental disorders, as well as many cancers^1,4,5^. Aberrant activation of FGFR3 signalling has a well-established role in the development of urothelial carcinoma (UC), and occurs as result of activating somatic mutations of *FGFR3*^6^, overexpression of the wildtype protein^7^, altered splicing^8^ and FGFR3 gene fusions^9^. *FGFR3* mutations are a very common occurrence in UC, particularly in low stage and grade tumours, reaching frequencies of over 80% in this subtype^10^. They are also thought to be an early change during urothelial transformation, as they are found in flat urothelial hyperplasia, a proposed precursor lesion for UC^11^. They occur at hotspots in exons 7 (codons 248 and 249), 10 (codons 372, 373, 375, 382, and 393) and 15 (codon 652)^12^ and cause constitutive activation of the receptor by either favouring the formation of disulfide or hydrogen bonds between adjacent monomers leading to receptor dimerization in the absence of ligand (exon 7 and 10 mutations)^13–15^, or by inducing conformational changes in the regulatory region of the receptor (exon 15 mutations)^16^. Overall, dysregulation of FGFR3 either through mutation, overexpression, or both occurs in around 80% of non-invasive and 54% of invasive UC^7^.

Numerous functional studies silencing or targeting mutant FGFR3 in bladder cancer cell lines have shown decreased cell proliferation and tumorigenic potential both *in vitro* and *in vivo*^17–20^. In view of the extensive evidence for a causative link between FGFR3 dysregulation and bladder tumorigenesis, and the high frequency of its alteration in UC, FGFR3 has been proposed as therapeutic target, and a number of inhibitors are currently in clinical trials^21^. However, further understanding of the signalling pathways activated by FGFR3 in normal and malignant urothelial cells is essential to support the use of FGFR3 inhibitors in the treatment of UC, and may also identify novel downstream targets for combination or second line therapy.

We have shown previously that FGFR3 activation in telomerase-immortalized normal human urothelial cells (NHUC) induces constitutive MAPK/ERK and PLCγ1 signalling and confers a proliferative and survival advantage at confluence, indicating that FGFR3-mutant cells can escape cell-cell contact inhibition of proliferation^22^. Gene expression profiling of NHUC expressing ectopic mutant FGFR3 identified ETV5, a member of the PEA3 subfamily of ETS transcription factors^23^, as a significantly upregulated gene compared to control NHUC (Geo submission GSE61352)^24^. Upregulation of ETV5 has been described in endometrial, ovarian and breast cancers, and chondrosarcomas, where it correlates with tumour infiltration and poor prognosis and regulates the expression of genes involved in epithelial-mesenchymal transition (EMT) and invasion^25–30^. Furthermore, ETV5 knockdown was shown to decrease malignant potential of mammary cancer cells^26^. The functional link between FGF signalling and PEA3 transcription factors has not yet been investigated during malignant transformation, but it has been described in embryonic development, where ETV4 and ETV5 are effectors of FGFs^31–33^. Therefore, in this study we investigate whether ETV5 and its downstream targets are crucial mediators of the oncogenic effects of mutant FGFR3 in urothelial cells.

## Results

### FGFR3 signalling modulates ETV5 expression via MAPK/ERK in normal urothelial cells

**Figure 1:**
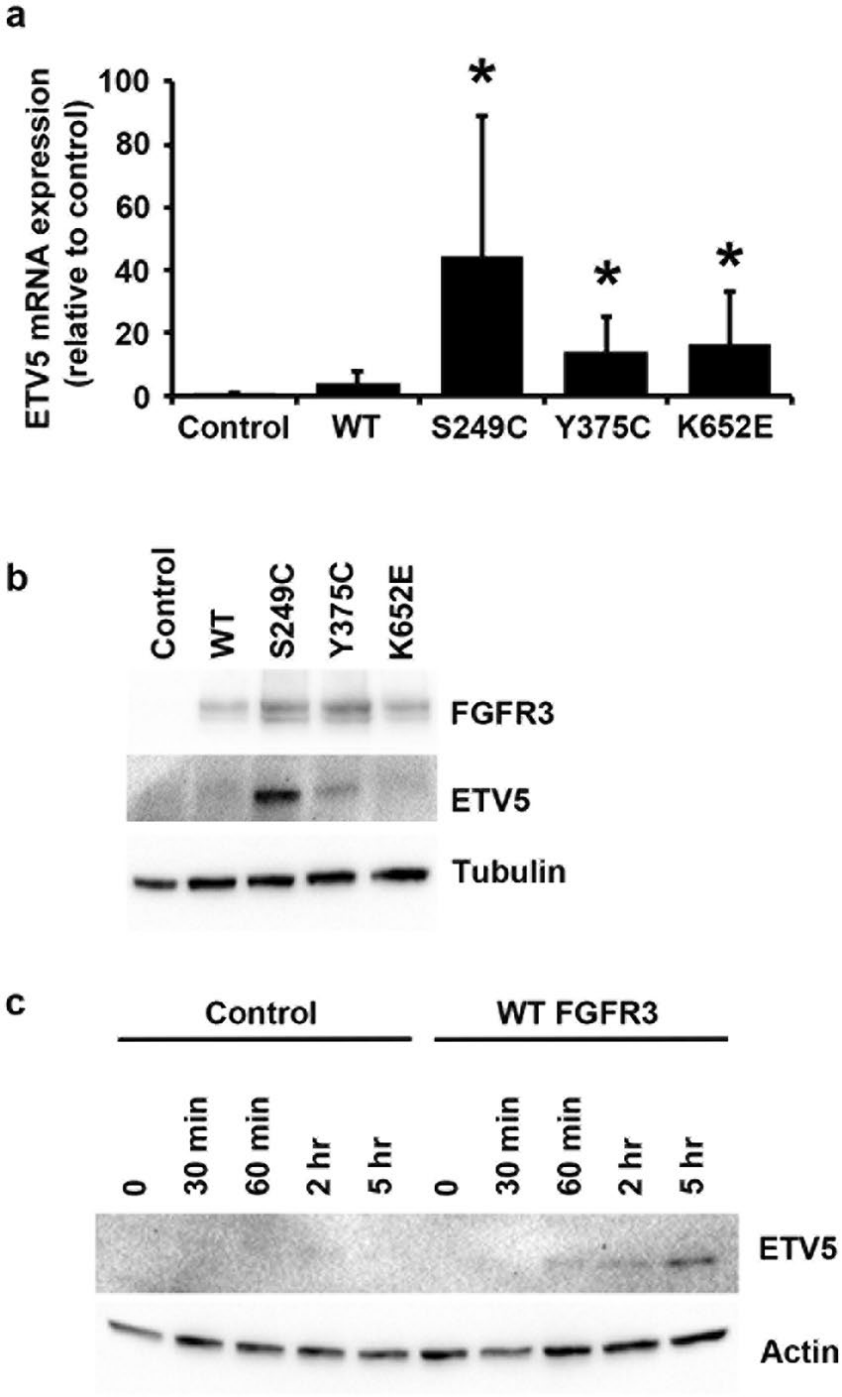
FGFR3-mediated regulation of ETV5 expression. (**a**) ETV5 mRNA and (**b**) ETV5 and FGFR3 protein levels in confluent TERT-NHUC with exogenous overexpression of wildtype (WT) or three types of mutant FGFR3 (S249C, Y375C, and K652E), and in control cells transduced with an empty vector (Control); (**c**) ETV5 protein levels following FGF1 treatment of TERT-NHUC overexpressing wildtype (WT) FGFR3 and of control cells transduced with the empty vector (Control). ETV5 mRNA was relatively quantified using Taqman Real-Time RT-PCR with SDHA as internal control, while ETV5 and FGFR3 proteins were visualized by western blotting with specific antibodies using alpha-tubulin or beta-actin as loading control. All experiments were repeated in triplicate. ‘*’ indicates a statistical significant difference compared with the control sample.

Gene expression profile data (Geo submission GSE61352)^24^ showed that ETV5 is a significantly upregulated gene in confluent telomerase-immortalized normal urothelial cells (TERT-NHUC) with ectopic expression of mutant (S249C) FGFR3. To confirm this, ETV5 mRNA levels were measured in confluent TERT-NHUC ectopically expressing three different FGFR3 mutant proteins (S249C, Y375C, and K652E). Expression of all three mutant forms of FGFR3 was accompanied by a significant upregulation of ETV5 mRNA (p = 0.05) (Fig. 1a). Protein upregulation was also observed in cells expressing S249C and Y375C FGFR3 (Fig. 1b). The magnitude of the effect was in the order S249C > Y375C > K652E, reflecting the previously observed mutation-dependent intensity of the phenotypic effects of mutant FGFR3 and the relative frequency of these mutations in tumours^22^. Upregulation of ETV5 protein was also observed in sub-confluent TERT-NHUC overexpressing exogenous wildtype FGFR3 following stimulation with the specific ligand, FGF1 (Fig. 1c).

**Figure 2:**
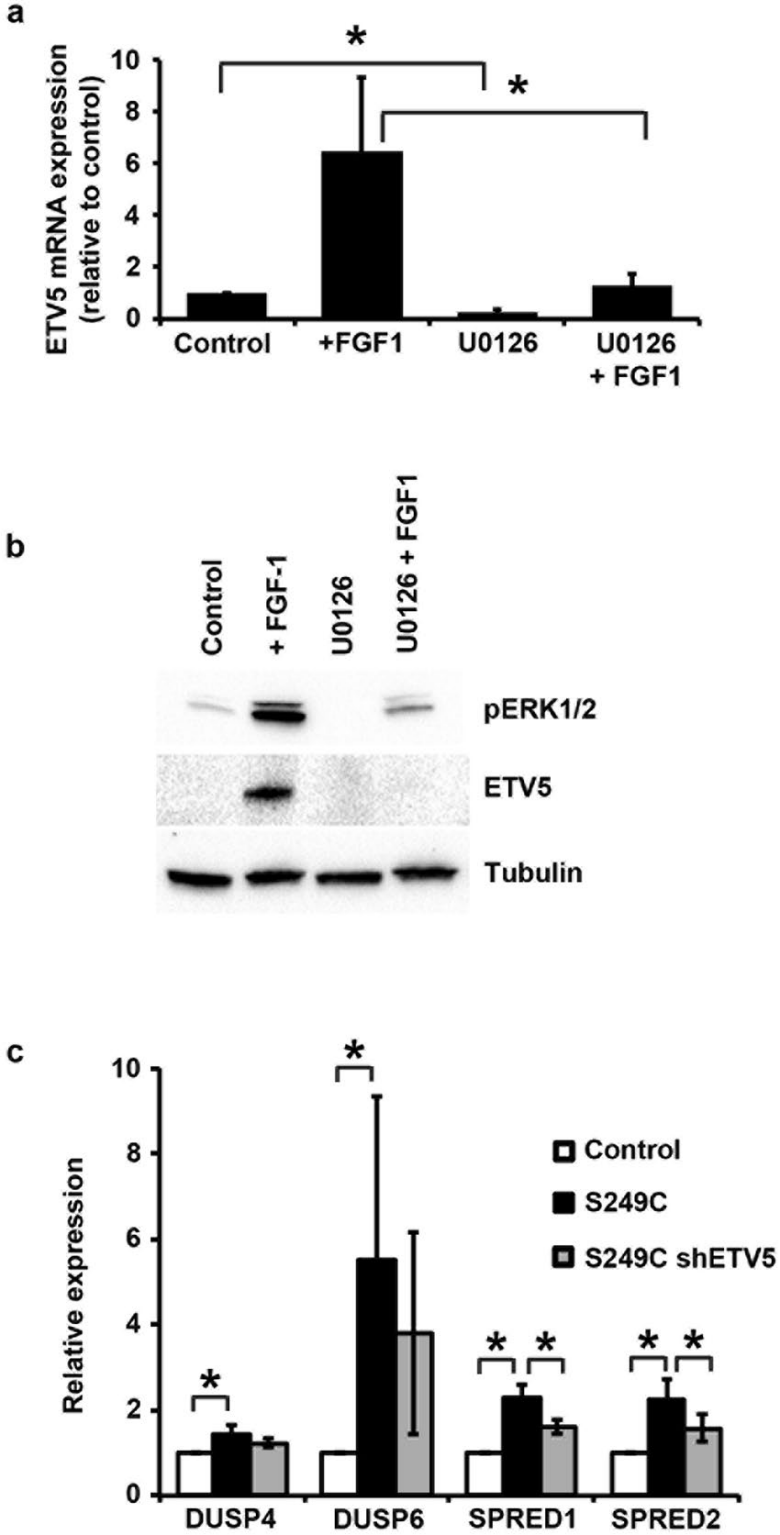
Regulation of ETV5 expression through MAPK/ERK. (**a**) ETV5 mRNA and (**b**) ETV5 and pERK1/2 protein levels in cells overexpressing wildtype FGFR3 stimulated for 5 hours with FGF1, with or without pre-treatment with the MAPK inhibitor U0126. ‘Control’ are unstimulated cells, ‘+ FGF1’ are cells stimulated with FGF1, ‘U0126’ are unstimulated cells treated with U0126, and ‘U0126 + FGF1’ are cells stimulated with FGF1 following 1 hour pre-treatment with U0126; (**c**) Expression of MAPK/ERK regulatory genes in TERT-NHUC with overexpressed mutant (S249C) FGFR3, compared with control cells transduced with the empty vector (Control) and cells with overexpressed S249C FGFR3 but silenced ETV5 (S249C shETV5). mRNA expression levels were relatively quantified using Taqman Real-Time RT-PCR with SDHA as internal control, while ETV5 and pERK1/2 proteins were visualized by western blotting with specific antibodies using alpha-tubulin as loading control. All experiments were repeated in triplicate. ETV5 silencing was performed using the sh155 hairpin. ‘*’ indicates a statistical significant difference between samples.

Since it has been reported that ETV5 is regulated by receptor tyrosine kinase signalling through MAPK/ERK in other cellular contexts^34^, we tested if this is also the case in telomerase-immortalized normal urothelial cells overexpressing wild type FGFR3. Exposure to the MAPK inhibitor U0126 reduced the basal level of ETV5 mRNA, and largely abolished FGF1-mediated ETV5 mRNA upregulation in these cells (Fig. 2a) (p = 0.05). Similarly, the upregulation of ETV5 protein after stimulation with FGF1 was blocked by U0126 pre-treatment (Fig. 2b).

Expression array data^24^ indicated that the expression of mutant FGFR3 in telomerase-immortalized normal urothelial cells can increase the level of MAPK/ERK pathway negative feedback regulator genes (DUSP4, DUSP6, SPRED1, SPRED2), which encode proteins limiting MAPK signalling by either dephosphorylating ERK1/2 or by preventing RAS activation^35^. Upregulation of these genes in TERT-NHUC expressing S249C FGFR3 was confirmed by Real Time RT-PCR (Fig. 2c) (p = 0.05). Interestingly, shRNA-mediated knockdown of ETV5 expression in these cells partially reversed upregulation of SPRED1 and SPRED2 (p = 0.05) (Fig. 2c).

From this we conclude that FGFR3 signalling can modulate ETV5 expression via MAPK/ERK in normal urothelial cells, and that ETV5 can in turn regulate genes involved in the MAPK/ERK negative feedback loop.

### Expression of ETV5 and cell density of normal urothelial cells are mutually dependent

**Figure 3:**
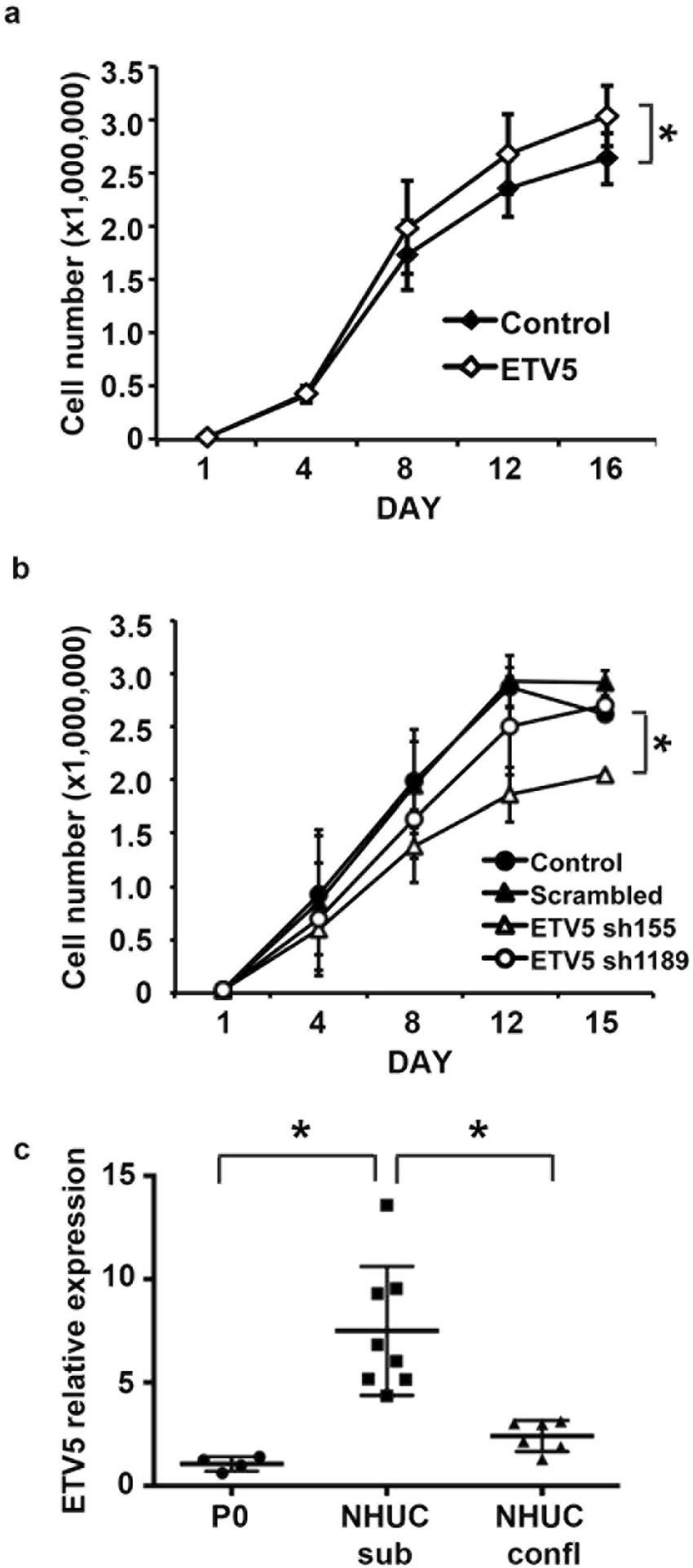
ETV5 expression and cell density of normal urothelial cells are mutually dependent. (**a**) Growth curves of TERT-NHUC overexpressing ETV5 and control cells transduced with the empty vector; (**b**) Growth curves of TERT-NHUC with knockdown of ETV5 (sh155 and sh1189), and control cells transduced with the empty vector (Control) or a scrambled shRNA; (**c**) mRNA relative expression of ETV5 in uncultured NHUC (passage zero, P0), and cultured mortal NHUC in sub-confluent (NHUC sub) and confluent conditions (NHUC confl). ETV5 mRNA expression levels were relatively quantified using Taqman Real-Time RT-PCR with SDHA as internal control. All experiments were repeated in triplicate. ‘*’ indicates a statistical significant difference between samples.

Previously we have shown that overexpression of mutant FGFR3 in TERT-NHUC results in increased proliferation and decreased apoptosis at confluence, leading to higher saturation density^22^. To investigate the role of ETV5 in mediating this phenotype we modulated its expression in these cells. A small but significant increase in saturation density was observed after ectopic expression of ETV5 in TERT-NHUC (p = 0.037) (Fig. 3a), similar to the effects previously described for mutant FGFR3^22^. Conversely, ETV5 knockdown in TERT-NHUC was associated with a decrease in cell proliferation in cells transfected with either of two shRNAs against ETV5, which reached significance with the most effective of the two hairpins (sh155, Supplementary Table S1) (p = 0.05) (Fig. 3b).

As the phenotypic effects associated with mutant FGFR3 in TERT-NHUC have been linked to confluent conditions, we measured ETV5 expression in mortal normal urothelial cells at confluence and sub-confluence. Interestingly, we found that ETV5 mRNA expression in sub-confluent NHUC is high but it drops significantly when these cells become confluent (p = 0.02), reaching levels closer to those observed in uncultured normal urothelial cells (P0) (Fig. 3c).

Overall these data indicate a reciprocal regulation of ETV5 levels, proliferation, and saturation density in normal urothelial cells.

### ETV5 contributes to the transformed phenotype of bladder cancer cells

To further investigate whether ETV5 upregulation contributes to the development of urothelial cancer, we used four established urothelial tumour lines. The UMUC14 cell line is derived from a transitional cell carcinoma of the renal pelvis and has the commonest FGFR3 mutation (S249C)^36^. The 97-7 cell line also has a S249C FGFR3 mutation but it is derived from an invasive T1 G2/3 bladder tumour and has a *TP53* mutation^37^. MGHU3 has a less common FGFR3 mutation (Y375C) and is derived from a low grade non-invasive bladder tumour^38^. Finally, CAL29 overexpresses wild type FGFR3, and is derived from an invasive T2G2 tumour^39^. Overall, these cell lines are representative of different tumour sub-types. UMUC14 and MGHU3 are representative of low grade and stage malignancies, in which FGFR3 mutation is more commonly found^7^, 97-7 is representative of FGFR3 mutant tumours with ability to invade the urothelial submucosa, and CAL29 is representative of the highest grade and stage tumours which often display FGFR3 upregulation rather than mutation^7^.

Firstly, we confirmed that mutant FGFR3 causes an increase in ETV5 expression in bladder cancer cells by using the two cell lines with the commonest FGFR3 mutation, 97-7 and UMUC14. Indeed, FGFR3 shRNA knockdown caused a significant decrease of ETV5 mRNA (Supplementary Fig. 1a,b) and protein (Supplementary Fig. 1c,d) expression in both cell lines. Notably, expression of ETV5 was restored when mutant (S249C) FGFR3 was re-expressed in 97-7 cells (p = 0.05) (Supplementary Fig. 1a,c).

**Figure 4:**
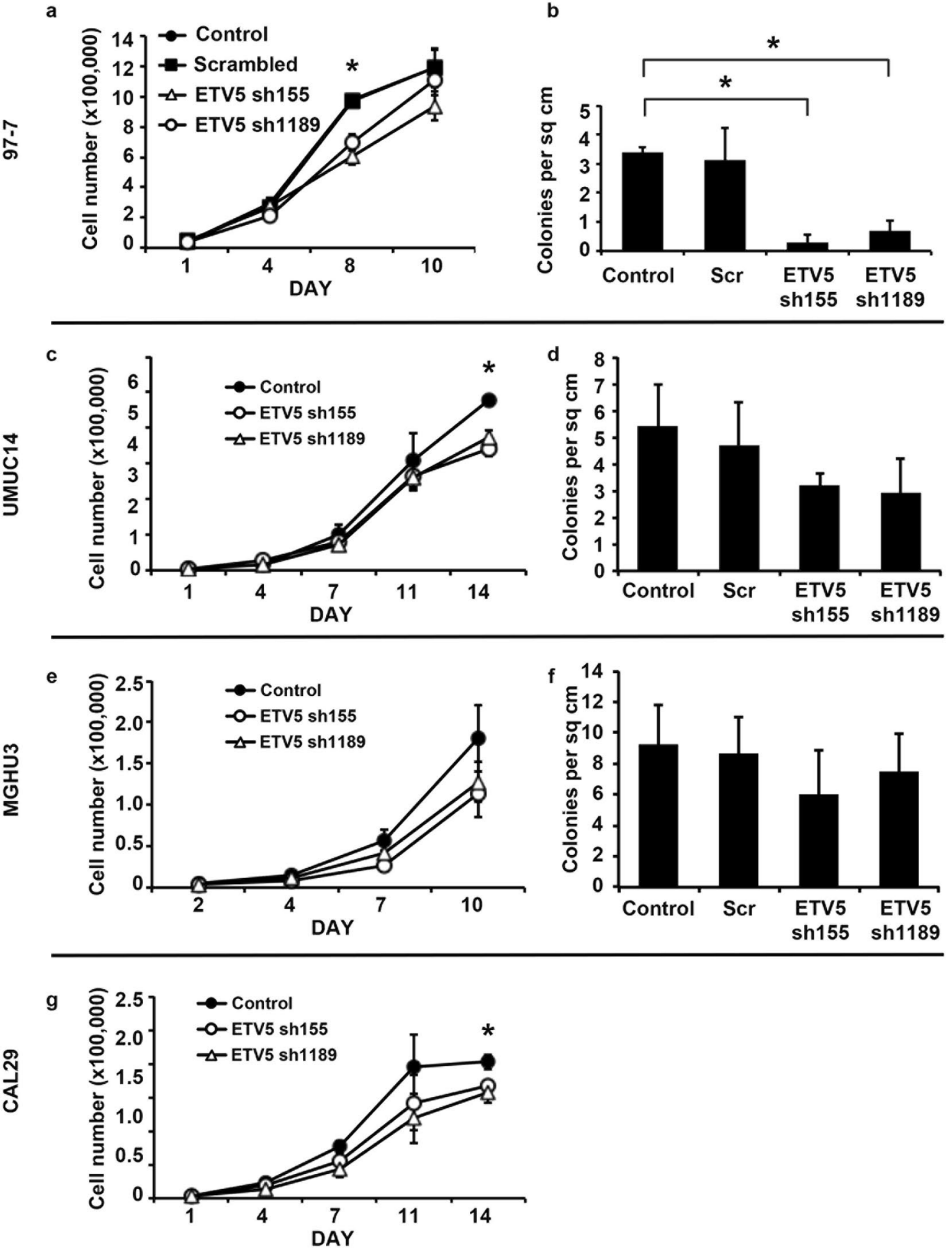
Effects of ETV5 knockdown in 97-7, UMUC14, MGHU3, and CAL29 cells. (**a**,**c**,**e**,**g**) Cell proliferation and (**b**,**d**,**f**) number of colonies formed in soft agar for UC cell lines with ETV5 knockdown (ETV5 sh155 and sh1189), compared with control cells transduced with an empty vector (Control) or a scrambled shRNA (Scr). The colony formation assay could not be performed in CAL29 cells as this cell line does not grow in soft agar. All experiments were repeated in triplicate. ‘*’ indicates a statistical significant difference between samples.

Subsequently, we looked at whether ETV5 knockdown mediates the oncogenic effect of mutant FGFR3 in cancer cells. We had previously shown that knockdown of endogenous mutant FGFR3 in the cell line 97-7 decreases cell proliferation and anchorage independent growth^18^. Therefore, we tested whether similar effects could be obtained by knocking down ETV5. When ETV5 expression was reduced with two different shRNAs, cell proliferation was significantly inhibited in 97-7 cells with both constructs (p = 0.05 on day 8) (Fig. 4a). Both shRNAs also decreased anchorage independent growth in these cells (p ≤ 0.05) (Fig. 4b). In the FGFR3-mutant MGHU3 and UMUC14 lines, which are derived from less invasive bladder tumours, ETV5 knockdown had a similar effect on reducing cell numbers at confluence (Fig. 4c,e), but a less striking effect on colony formation (Fig. 4d,f). Finally, in the high grade/stage wild type line, CAL29, knockdown of ETV5 significantly reduced cell proliferation (p = 0.05 on day 14) (Fig. 4g). The effect on anchorage independent growth could not be tested, as these cells do not form colonies in soft agar. Interestingly, when we checked the expression of the EMT-related ETV5 target genes MMP2, FOXM1 and NID1 after ETV5 silencing, a significant decrease was observed in the invasive 97-7 cells (Supplementary Fig. S2). A small but significant (p = 0.05) decrease in NID1 was also observed in the invasive CAL29 cells after ETV5 silencing, although no change in FOXM1 and a significant (p = 0.05) increase in MMP2 expression was seen in these cells (Supplementary Fig. S2). In the non-invasive UMUC14 and MGHU3 lines, no change in NID1 and FOXM1 expression was detected after ETV5 silencing, whilst an increase in MMP2 expression was seen in UMUC14 (Supplementary Fig. S2). This suggests that the effect of ETV5 upregulation in bladder cells may differ depending on tumour sub-type.

Overall therefore, our results show that ETV5 confers a proliferative advantage to bladder cancer cells. They also suggest that ETV5 may mediate anchorage independent growth and the expression of genes involved in invasion and EMT in the subset of more invasive FGFR3-mutant tumours.

### FGFR3 signalling modulates the expression of TAZ in urothelial cells via ETV5

**Figure 5:**
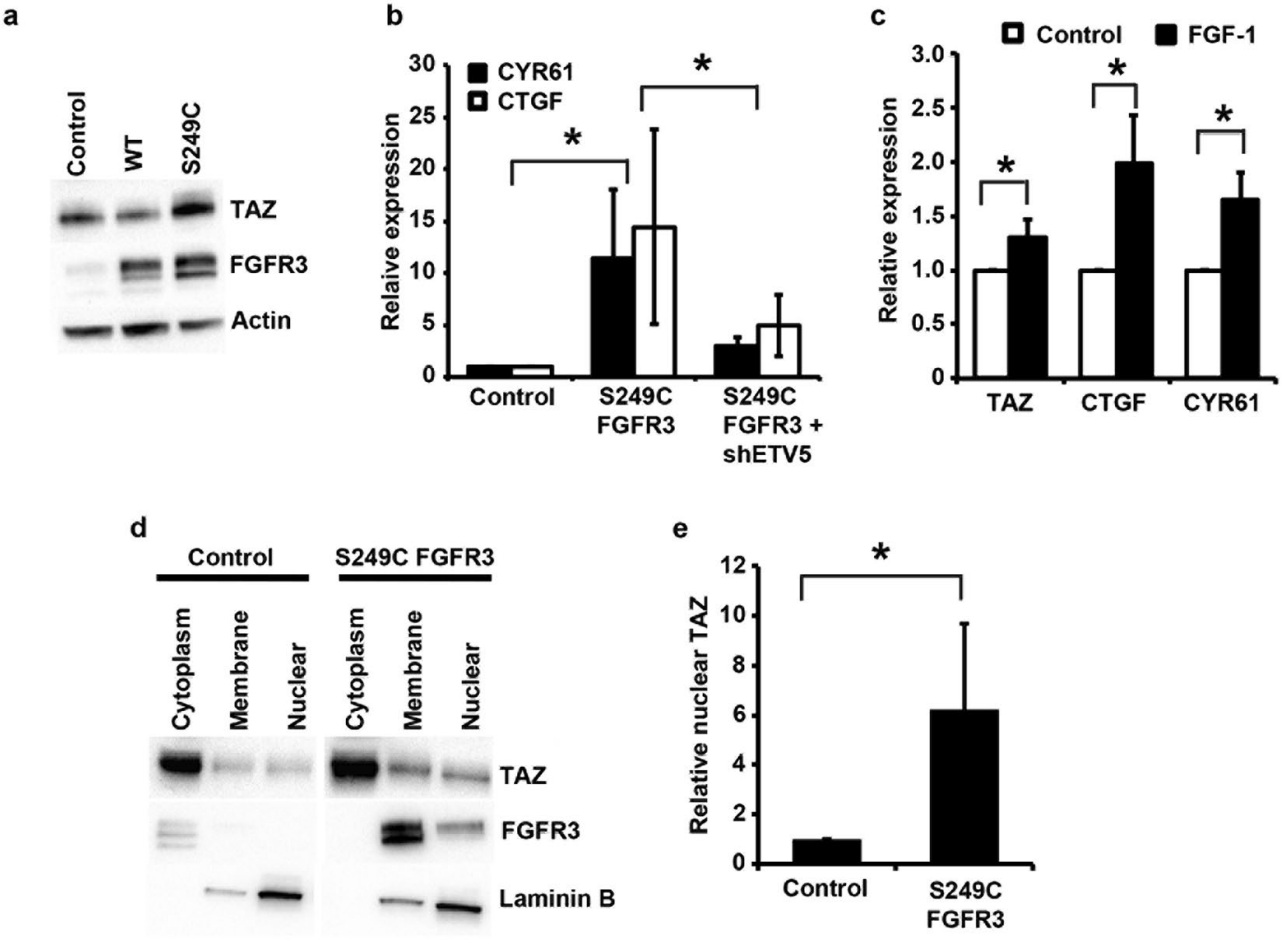
TAZ activity is altered in urothelial cells after FGFR3 or ETV5 modulation. (**a**) TAZ and FGFR3 protein expression in confluent TERT-NHUC cells overexpressing wildtype (WT) and S249C FGFR3, and in cells transduced with the empty vector (control); (**b**) CTGF and CYR61 levels in confluent TERT-NHUC expressing the S249C FGFR3 mutation (S249C), cells expressing the S249C FGFR3 mutation but with ETV5 knockdown (S249C shETV5), and cells transduced with the empty vector (Control); (**c**) TAZ, CTGF and CYR61 mRNA expression in sub-confluent TERT-NHUC overexpressing wild type FGFR3 five hours after stimulation with FGF1, compared to unstimulated control cells; (**d**) Representative blot showing TAZ protein levels in cytoplasmic, membrane and nuclear sub-fractions of confluent TERT-NHUC cells overexpressing S249C FGFR3 and control cells transduced with the empty vector; (**e**) Quantification of TAZ nuclear protein levels in confluent TERT-NHUC overexpressing S249C FGFR3 relative to control cells transduced with the empty vector. Values were adjusted for overall TAZ expression (calculated as the sum of expression in the different cellular fractions), to account for the higher overall expression in FGFR3 mutant cells. mRNA expression levels were relatively quantified using Taqman Real-Time RT-PCR with SDHA as internal control, while TAZ protein level was visualized by western blotting with a specific antibody, using actin or laminin B1 as loading or fractionation control. All experiments were repeated in triplicate. ‘*’ indicates a statistically significant difference between samples.

To identify urothelial-specific downstream targets of ETV5, we performed gene expression array analysis of confluent 97-7 cells with and without ETV5 knockdown (Geo submission GSE122306). A total of 91 genes significantly modulated above the 1.5-fold threshold were identified (Supplementary Table S2). One of the top 10 most significantly down-regulated hits in cells with ETV5 knockdown was TAZ (WWTR1), which was also upregulated about 2-fold in confluent telomerase-immortalized normal urothelial cells expressing mutant FGFR3 compared to control cells (GEO submission GSE61352)^24^. TAZ is a co-transcriptional regulator, which together with the highly homologous protein YAP1, mediates the transcription of pro-proliferative and anti-apoptotic genes, and upregulation is observed in many cancers^40^. Nuclear localization and activation of TAZ and YAP1 is regulated by phosphorylation through the Hippo pathway, a tightly regulated and conserved kinase cascade, based on cues triggered by cell-cell contact and cell density^41,42^. In contact-inhibited cells, the Hippo pathway is active, leading to the phosphorylation, cytoplasmic retention and degradation of YAP1 and TAZ, limiting their activity^43,44^. Consequently, upregulated TAZ represents a biologically plausible explanation for the escape from cell-cell and cell-contact inhibition and higher proliferation at confluence associated with mutant FGFR3. Therefore we investigated this further. A 1.9-fold upregulation of TAZ mRNA expression was confirmed in TERT-NHUC expressing S249C FGFR3 compared with control cells (p = 0.05), and a corresponding 2-fold upregulation was observed at the protein level (p = 0.04) (Fig. 5a). Consistently, a significant increase in mRNA level of two *bona fide* TAZ transcriptional targets, CTGF and CYR61^45^, was observed in confluent TERT-NHUC cells expressing mutant FGFR3 compared to confluent control cells (p = 0.037). Notably, this upregulation was reversed upon ETV5 knockdown in these cells (Fig. 5b). To check that the differences in TAZ levels were not due to differences in cell density at confluence, and to further confirm that FGFR3 signalling can increase TAZ mRNA expression, sub-confluent TERT-NHUC over-expressing exogenous wildtype FGFR3 were stimulated with FGF1. A small but significant increase in the expression of TAZ and its target genes, CTGF and CYR61, was also observed after FGF1 stimulation in sub-confluent conditions (Fig. 5c) (p = 0.037).

TAZ co-transcriptional activity requires nuclear localization. Therefore, we investigated TAZ subcellular localization in isogenic TERT-NHUC with and without expression of mutant FGFR3. Results show that cells expressing mutant FGFR3, after correction for the increased TAZ expression, have an increase amount of nuclear TAZ compared with controls (Fig. 5d,e). In cells with mutant FGFR3, 33–40% of TAZ was localized in the nucleus, compared to 9–14% in control cells (p = 0.05). Interestingly, sub-cellular fractionation also showed that control TERT-NHUC have very low levels of endogenous FGFR3 and this is localized in the cytoplasm. However, most S249C FGFR3 is located at the membrane, with a small fraction located in the nucleus. The significance of this is unclear at this stage, but it is in line with previous reports showing nuclear translocation of activated FGFRs^46–48^.

Overall these data show that mutant FGFR3 upregulates ETV5 in urothelial cells, which in turn causes an increase in nuclear TAZ, leading to hyperproliferation in confluent conditions.

### The expression of ETV5 and TAZ is correlated in bladder tumours

To investigate the link between FGFR3, ETV5 and TAZ *in vivo* we interrogated two large publically available bladder tumour data sets^49,50^. In the Sjodahl *et al.* study, including 308 tumours of which the majority (n = 216) were low stage (Ta-T1)^49^, no information is available about FGFR3 mutation status. Therefore we used FGFR3 expression as surrogate of mutation, as most mutant tumours also have FGFR3 upregulation^7^. No correlation was observed between FGFR3 and ETV5 expression levels in this study. In the TCGA study, which includes 408 high stage (T2-T4) tumours^50^ for which information on FGFR3 mutation status is available, no significant difference in ETV5 expression was observed between mutant and wild type tumours. This is not surprising as we have shown that ETV5 expression is modulated by MAPK/ERK signalling, which is a point of convergence of many signalling pathways altered in bladder cancer, such as PI3K, EGFR, and RAS. Therefore other molecular changes may drive an upregulation of ETV5 in FGFR3-wild type tumours. However, a trend to a positive correlation between ETV5 and TAZ expression level was found in both studies (R = 0.370, p = 12e-14 in the TCGA study; R = 0.181–0.229, p = 1.5e-03-4.8e-5 in the Sjodahl *et al*. study, depending on the probes selected) (Supplementary Fig. S3). Notably, a trend to a positive correlation was also found between the levels of ETV5 and the TAZ target genes, CYR61 and CTGF, in both studies (CYR61: R = 0.251 and p = 2.9e-07 in TCGA study, R = 0.161–0.320 and p = 4.6e-03-9.2e-09 in the Sjodhal *et al.* study, depending on the probes selected; CTGF: R = 0.302 and p = 4.5e-10 in TCGA study, R = 0.183–0.440 and p = 1.2e-03-5.2e-16 in the Sjodhal *et al*. study, depending on the probes selected) (Supplementary Fig. S3). This confirms a link between ETV5 expression and the level of TAZ and its target genes in bladder tumours.

## Discussion

Members of the ETS-family of transcription factors are downstream effectors of FGF-signalling during embryonic development^31–33^ but their role in urothelial carcinomas driven by FGFR3 mutation has not been investigated so far. Our results substantiate ETV5 as a downstream target of FGFR3 signalling in urothelial cells. ETV5 mRNA and protein levels are constitutively upregulated after expression of mutant FGFR3 in telomerase-immortalized normal urothelial cells, and interestingly the intensity of upregulation is consistent with our previous observation of a hierarchy in the potency of different FGFR3 mutations which reflects their relative frequency in patients (S249C > Y375C > K652E)^22^. ETV5 expression is also increased in telomerase-immortalized normal urothelial cells expressing wildtype FGFR3 shortly after stimulation with the FGFR3 specific ligand, FGF1. Consistently, knockdown of endogenous mutant FGFR3 in UC cell lines is followed by ETV5 downregulation, whilst ETV5 levels are restored after re-expression of mutant FGFR3. Notably, ETV5 was one of the genes significantly downregulated after FGFR3 silencing in the bladder cancer cell line RT112^51^, or after treatment of cancer cell lines with the FGFR inhibitor AZD4547^52^, independently confirming our results.

We also show that FGFR3-induced upregulation of ETV5 is mediated *via* MAPK/ERK signalling, as it is blocked by treatment with the MAPK inhibitor, UO126. This suggests that the role of ETV5 in bladder carcinogenesis may not be limited to FGFR3-mutant tumours but may also be relevant to tumours with other alterations resulting in MAPK/ERK activation, such as RAS mutations. Indeed, in BRAF mutant melanoma and colon cancer cell lines ETV5 was included in a 52-gene ERK-signalling output signature^34^.

Our data show that ETV5 is important in maintaining the malignant phenotype of bladder cancer cells, and that its knockdown can recapitulate some of the phenotypic effects of FGFR3 knockdown in UC cells, such as decreased cell proliferation and reduced anchorage independent growth^7^. However, the consequences of ETV5 silencing appeared to be less intense than those previously described after knockdown of FGFR3 in these cells. This is not surprising, as other proteins may be important in mediating FGFR3 signalling in addition to ETV5. For example, we found that ETV1, another member of the ETS-family with high homology of the DNA binding domain, and significant overlap in promoter binding specificity^23^, was also upregulated in normal urothelial cells expressing mutant FGFR3 (unpublished data). Therefore it is possible that knockdown of only one member of the family may lead to limited phenotypic effect due its redundancy and that combined knockdown of additional members may have more pronounced effects.

As ETS-family transcription factors have not been studied in relation to bladder cancer so far, little is known about their transcriptional targets in the context of urothelial cells. In other tumour types, ETV5 has been found to mediate EMT through modulation of invasion-related genes, such as NID1, MMP2 and FOXM1^30,53,54^. Similarly, these genes were also modulated by ETV5 in the invasive bladder cancer cell line 97-7, but not in the non-invasive FGFR3-mutant cell lines, MGHU3 and UMUC14. As FGFR3 mutations are more common in low grade and stage bladder tumours, and are an early change during urothelial transformation, it is likely that besides favouring EMT in more advanced tumours, ETV5 may modulate other classes of genes in early neoplastic lesions. Identification of these genes may shed further light on the molecular events driving bladder tumour development, and their inhibition may offer novel therapeutic approaches in the treatment of this disease.

We have shown previously that expression of mutant FGFR3 in TERT-NHUC is associated with increased cell density at confluence^22^, and we hypothesized that this may favour pathogenesis by leading to hyperplastic pre-malignant lesions in the bladder. In this study we identify a cross-talk between FGFR3 signalling and the Hippo pathway, which has a crucial role in regulating cell proliferation, death and differentiation based on cues triggered by cell-cell contact and cell density^41,44^. Our *in vitro* results show that activation of FGFR3 signalling in urothelial cells alters the expression of TAZ and two of its *bona fide* targets *via* ETV5, and are independently supported by the finding of an *in vivo* positive correlation between ETV5 and TAZ expression in two large publically available gene expression datasets from bladder tumours. ETV5 could regulate TAZ expression through different mechanisms, such as directly inducing its transcription, or regulating the transcription of genes involved in extracellular matrix remodelling and stiffness, which have been shown to control TAZ activation and nuclear localization^55^. Our preliminary data suggest that several consensus regions for ETV5 binding are found in the TAZ promoter (data not shown). Whilst this manuscript was under review, it has been reported that indeed ETV1, ETV4 and ETV5 can transcriptionally upregulate TAZ in prostate cancer cells^56^. To our knowledge this is the first report of a link between mutant FGFR3 and TAZ, although notably a recent study has indicated that FGFR signalling in cholangiocarcinoma cell lines induces the expression and nuclear localization of YAP1^57^. Dysregulation of the Hippo pathway and over-activation of YAP1 and TAZ is common in cancer, and is involved in invasion and metastasis^40^. In bladder cancer the role of the Hippo pathway has not been thoroughly investigated, but over-expression of YAP1 was shown to be common in UC and associated with poorer prognosis^58^.

In conclusion, this is the first comprehensive investigation of the role of ETV5 in bladder cancer. We have shown that ETV5 is a downstream target of mutant FGFR3 through the MAPK/ERK signalling pathway, is involved in the cross-talk between FGFR3 signalling and the Hippo pathway, upregulates the expression of genes involved in EMT in cell derived from invasive tumours, and mediates proliferation and anchorage-independent growth of bladder cancer cells. As the Hippo pathway plays an emerging key role in cancer and its pharmacological inhibition in cell lines from other tumour types has shown promising results^59^, the link between ETV5 and TAZ, and the potential of TAZ as therapeutic target in urothelial carcinoma deserves further investigation.

## Materials and Methods

### Cell lines

Normal urothelial cells (NHUC) were established in our laboratory from primary uncultured normal urothelium (passage zero, P0) obtained from human ureters removed at nephrectomy from patients with renal carcinoma^60^, and immortalized with telomerase (TERT-NHUC)^61^. Cancer cell lines were obtained either from the laboratory of origin or a recognized cell repository and authenticated by DNA profiling using the PowerPlex® 16 kit (Promega UK, Southampton, UK). All cell lines were cultured at 37 °C in 5% CO_2_, either in Keratinocyte Growth Medium 2 containing 0.09 mM CaCl_2_ (Promocell, Heidelberg, Germany) (TERT-NHUC) or in standard growth media (cancer lines). For FGF1 stimulation, TERT-NHUC were incubated in Keratinocyte Growth Medium depleted in growth factors for one hour with or without 10 µM U0126, followed by treatment with 20 ng/ml recombinant human FGF1 and 10 μg/ml heparin.

### Modulation of ETV5 expression

Knockdown of FGFR3 and ETV5 was performed by shRNA as detailed previously^18^. Oligonucleotide sequences targeting FGFR3 and the scrambled control sequence were as published^18^. For ETV5, oligonucleotide sequences were designed to produce hairpins targeting different sites along the mRNA. Oligonucleotides were annealed and cloned into a retroviral expression vector containing a puromycin resistance cassette (pRetroSuper-puro). The magnitude and persistence of ETV5 knockdown were tested over three weeks. Two hairpins (sh155, Top strand 5′ CTTCAAGAGGCTTGGTTAGTTCAAGAGACTAACCAAGCCTCTTGAAGTTTTTTGGGCC3′, Bottom strand 5′CAAAAAACTTCAAGAGGCTTGGTTAGTCTCTTGAACTAACCAAGCCTCTTGAAG3′; and sh1189, Top strand 5′GCTGATAGAACCGGAAGAGTTCAAGAGACTCTTCCGGTTCTATCAGCTTTTTTGGGCC3′, bottom strand 5′CAAAAAAGCTGATAGAACCGGAAGAGTCTCTTGAACTCTTCCGGTTCTATCAGC3′) which produced stable knockdown >50% in 97-7 cells were selected for subsequent experiments. Levels of ETV5 knockdown achieved with these two hairpins in the various cell lines used in the study are summarized in Supplementary Table S1. For phenotypic assays, both hairpins were used. Real-Time RT-PCR assessment of expression of downstream genes was only performed for the sh155 hairpin, which produced the greatest knockdown.

For ETV5 overexpression, the coding sequence for human ETV5 (Addgene, Cambridge, US), was subcloned into a retroviral expression vector (pFB; Stratagene, La Jolla, CA) modified to contain a hygromycin resistance cassette (pFB-hyg). pFB-hyg vectors containing wildtype or three different mutant forms of FGFR3 (S249C, Y375C and K652E) were produced as previously described^22^.

pFB-hyg or pRetroSuper-puro plasmids were transfected into Phoenix A packaging cells using TransIT^®^−293 (Cambridge BioScience, Cambridge, UK), medium was harvested after 48 hr, filtered with 0.45 µm filters, and mixed with hexadimethrine bromide to a final concentration of 8 µg/ml. Urothelial cell lines were exposed to viral supernatants for 8 hours for two consecutive days. Successfully transduced cells were selected using puromycin or hygromycin, as appropriate.

### Phenotypic *in vitro* assays

Phenotypic assays were carried out within 30 population doublings of transduction and repeated 3 times. For growth curves, 3 × 10^4^ cells were seeded in triplicate wells in 6-well plates and counted on day 1 to 14. To test anchorage independent growth, 3–5 × 10^4^ cells were cultured in triplicate wells in 6-well plates in medium containing 0.3% agarose, with weekly feeding. Colonies bigger than 100 μm were counted on day 28, after staining with 8 mM p-iodonitrotetrazolium violet.

### RNA extraction and expression analysis

RNA from cell lines was extracted using TRIzol® reagent (Invitrogen, UK), DNAse treated, and re-purified using the RNeasy kit (Qiagen Ltd., Crawley, UK). cDNA synthesis and Taqman^®^ Real-Time PCR were performed as described previously^62^, using the following TaqMan^®^ Gene Expression Assays (Applied Biosystems, Warrington, UK): Hs00188166_m1 (*SDHA*, internal control), Hs927557_m1 (*ETV5*), Hs00159600_m1 (*NID1*), Hs01073586_m1 (*FOXM1*), Hs01548727_m1 (*MMP2*), Hs210007_m1 (TAZ), Hs155479_m1 (CYR61), and Hs1026927_g1 (CTGF). Gene expression was normalized to the internal control using the ΔCt method and was quantified relative to a positive control sample. Non-template negative controls were included in each plate.

### Protein extraction and western blotting

Total protein extraction and quantification was carried out as previously described^8^. Forty µg of heat-denatured proteins were separated by electrophoresis using 10% Mini-PROTEAN® TGX Stain-Free™ Precast Gels (Bio-Rad Laboratories Ltd., Hempstead, UK). Separated proteins were transferred onto a Trans-Blot® Turbo™ Midi Nitrocellulose membrane (Bio-Rad Laboratories Ltd.), and incubated with 1:1000 primary antibodies (anti-ETV5, clone 3B10, Novus Biologicals Ltd, Cambridge, UK; anti-FGFR3, sc-13121, Insight Biotechnology Ltd, Middlesex; anti-phospho-ERK1/2 (Tyr 204), sc-7383, Insight Biotechnology Ltd, Middlesex; anti-YAP/TAZ, D24E4, Cell Signalling Technology Inc.; anti-Laminin B1, Ab16048, Abcam, Cambridge, UK; anti-beta-actin, sc-81178, Insight Biotechnology Ltd; anti-tubulin alpha, MorphoSys UK Ltd., Kidlington, UK) overnight at 4 °C. Bound primary antibody was detected using HRP-conjugated secondary antibody and Luminata Forte Western HRP Substrate (Millipore UK Limited, Watford, UK). Extraction of proteins from specific subcellular fractionation was performed using the Subcellular Protein Fractionation Kit for Cultured Cells (Fisher Scientific Ltd., Loughborough).

### Gene expression profiling and analysis

Gene expression profiling was performed as previously described^24^ by the Cancer Research UK Manchester Institute Microarray Service. Briefly, DNAse treated RNA was used to prepare biotinylated target cRNA, following the Affymetrix One Cycle Target Preparation Protocol, which was then hybridized on Affymetrix HG U133 Plus 2.0 oligonucleotide arrays (Affymetrix UK Ltd.). Expression data were analysed with Partek Genomic Suite 6.5. Raw values of intensity were normalized using RMA, and significant differences in expression levels between samples were identified using t-test, after correction for multiple testing.

### Statistical analysis

Significant differences in gene expression levels, cell number, and colony number between isogenic cell lines were assessed using the Mann-Whitney test, with the SPSS 12.0 statistical analysis software (SPSS Inc., Chicago, US). A p ≤ 0.05 was accepted as significant. All experiments were repeated at least three times, and error bars show standard deviation between replicate experiments.

## Electronic supplementary material

Supplementary data
