# Supplementary material for: ETV5 links the FGFR3 and Hippo signalling pathways in bladder cancer
Source: Sci Rep. 2019 Apr 5;9:5740. doi: 10.1038/s41598-018-36456-3 (PMC6450944; doi:10.1038/s41598-018-36456-3)
Supplement: Supplementary file 1 — Supplementary data [file 41598_2018_36456_MOESM1_ESM.pdf]

ETV5 links the FGFR3 and Hippo signalling pathways in bladder cancer

Erica di Martino, Olivia Alder, Carolyn D. Hurst and Margaret A. Knowles

Supplementary Table S1. ETV5 knockdown levels achieved in different cell lines. Relative ETV5 expression in cells transduced with two different shRNA against ETV5 (sh155 and sh1189), or cells transduced with a scrambled shRNA, compared with control cells transduced with the empty vector. Sh155 achieved the best knockdown and was associated with the strongest phenotypic changes.

	**Control**	**Scrambled**	**sh155**	**sh1189**
**TERT-NHUC**	1	0.82	0.21	0.48
**97-7**	1	0.80	0.07	0.13
**MGHU3**	1	1.32	0.49	0.65
**CAL29**	1	1.19	0.12	0.30
**UMUC14**	1	1.15	0.30	0.43

Supplementary Table S2. Genes significantly up- and down-regulated in 97-7 cells with ETV5 knockdown. Gene expression arrays were performed in triplicate for control cells transduced with empty vector, cells transduced with a scrambled shRNA, and cells transduced with one of two anti-ETV5 shRNAs (sh155 and sh1189). For the analysis, samples were divided into two groups: control cells and cells transfected with scrambled shRNA (control group, n=6), and cells transfected with either shRNA (shRNA group, n=6). Mean ratio between the shRNA and the control groups, and associated p value is reported for differentially expressed genes.

Mean Ratio (shRNA/control)	p-value	Gene Symbol	Gene Title
0.30	4.10E-07	ETV5	ets variant 5
0.39	2.23E-06	ETV5	ets variant 5
0.20	1.25E-05	ETV5	Ets variant 5
0.17	3.65E-05	ETV5	ets variant 5
0.61	5.87E-05	HIP1	Huntingtin interacting protein 1
0.17	8.74E-05	ETV5	Ets variant 5
0.66	0.000157	CHST11	Carbohydrate (chondroitin 4) sulfotransferase 11
0.66	0.000281	PECAM1	platelet/endothelial cell adhesion molecule
0.64	0.000714	DACT1	dapper, antagonist of beta-catenin, homolog 1 (Xenopus laevis)
0.58	0.000801	WWTR1	WW domain containing transcription regulator 1
0.66	0.000812	PPM1H	protein phosphatase 1H (PP2C domain containing)
0.37	0.000844	VCAN	versican
0.56	0.001058	HIP1	huntingtin interacting protein 1
0.63	0.001116	KLF15	Kruppel-like factor 15
0.41	0.001406	SEPP1	selenoprotein P, plasma, 1
0.60	0.001543	PTX3	pentraxin-related gene, rapidly induced by IL-1 beta
0.66	0.001676	TGM2	transglutaminase 2
0.65	0.001721	NRCAM	neuronal cell adhesion molecule
0.61	0.001724	SEC22A	SEC22 vesicle trafficking protein homolog A (S. cerevisiae)
0.65	0.0019	LOC150051	hypothetical LOC150051
0.60	0.001912	FN1	fibronectin 1
0.63	0.001995	CHST11	Carbohydrate (chondroitin 4) sulfotransferase 11
0.61	0.002121	SERPINA1	serpin peptidase inhibitor, clade A, member 1
0.53	0.002651	MAP7D2	MAP7 domain containing 2
0.64	0.002698	SERPINA1	serpin peptidase inhibitor, clade A, member 1
0.60	0.002806	NDN	necdin homolog (mouse)
0.60	0.00361	CAMK2B	calcium/calmodulin-dependent protein kinase II beta
0.67	0.006253	FHOD3	formin homology 2 domain containing 3
0.52	0.007502	FAM65B	family with sequence similarity 65, member B
0.64	0.010353	FN1	fibronectin 1
0.65	0.010421	LPL	lipoprotein lipase
0.48	0.010918	AZGP1	alpha-2-glycoprotein 1, zinc-binding
0.64	0.011721	EPDR1	ependymin related protein 1 (zebrafish)
0.58	0.012258	DNAJC15	DnaJ (Hsp40) homolog, subfamily C, member 15
0.63	0.012993	FN1	fibronectin 1
0.63	0.013036	FN1	fibronectin 1
0.58	0.013602	EPSTI1	epithelial stromal interaction 1 (breast)
0.63	0.013819	FN1	fibronectin 1

0.66	0.014487	HIP1	huntingtin interacting protein 1
0.60	0.015607	KCNK5	potassium channel, subfamily K, member 5
0.66	0.015904	AZGP1	alpha-2-glycoprotein 1, zinc-binding
0.65	0.016848	HIST1H4B	Histone cluster 1, H4b
0.63	0.020372	CYLD	cylindromatosis (turban tumor syndrome)
0.58	0.020976	DCLK1	doublecortin-like kinase 1
0.65	0.021968	ZNF585A	zinc finger protein 585A
0.66	0.022417	RUNX1	runt-related transcription factor 1
0.62	0.024915	DPY19L2	dpy-19-like 2 (C. elegans)
0.62	0.029795	TFCP2L1	transcription factor CP2-like 1
0.49	0.033777	PS1TP4	HBV preS1-transactivated protein 4
0.61	0.035881	RC3H2	ring finger and CCCH-type zinc finger domains 2
0.57	0.040383	TFCP2L1	transcription factor CP2-like 1
0.63	0.041299	VSTM2L	V-set and transmembrane domain containing 2 like
0.62	0.044441	ECOP	EGFR-coamplified and overexpressed protein
0.62	0.046025	SLC8A1	solute carrier family 8 (sodium/calcium exchanger), member 1
1.68	8.35E-05	SERPINB13	serpin peptidase inhibitor, clade B (ovalbumin), member 13
1.79	0.000121	RNF182	ring finger protein 182
1.76	0.000181	IGFL1	IGF-like family member 1
1.50	0.000295	D4S234E	DNA segment on chromosome 4 (unique) 234 expressed sequence
2.06	0.000322	SESN3	sestrin 3
1.54	0.000356	FAM84A	Family with sequence similarity 84, member A
1.96	0.000459	CAPNS2	calpain, small subunit 2
1.51	0.000618	LEPREL1	leprecan-like 1
1.76	0.000735	SERPINB13	serpin peptidase inhibitor, clade B (ovalbumin), member 13
1.53	0.000856	PPP2R2C	protein phosphatase 2, regulatory subunit B, gamma isoform
1.57	0.000959	CTSL2	cathepsin L2
1.51	0.001073	GLRX	glutaredoxin (thioltransferase)
1.57	0.001128	AKR1B10	aldo-keto reductase family 1, member B10 (aldose reductase)
1.62	0.001272	BTG2	BTG family, member 2
1.64	0.001341	FGFBP1	fibroblast growth factor binding protein 1
1.67	0.001398	SERPINB13	serpin peptidase inhibitor, clade B (ovalbumin), member 13
1.86	0.001714	S100A7	S100 calcium binding protein A7
1.51	0.00182	HSD11B2	hydroxysteroid (11-beta) dehydrogenase 2
1.65	0.002111	KRT24	keratin 24
1.78	0.002448	HES2	hairy and enhancer of split 2 (Drosophila)
1.53	0.00258	COX7B2	cytochrome c oxidase subunit VIIb2
1.75	0.003105	SERPINB13	serpin peptidase inhibitor, clade B (ovalbumin), member 13
2.08	0.00367	FABP4	fatty acid binding protein 4, adipocyte
1.59	0.00387	LYPD3	LY6/PLAUR domain containing 3
1.52	0.004128	FAM84A	family with sequence similarity 84, member A
1.70	0.00414	CRYAB	crystallin, alpha B
1.90	0.005649	SESN3	sestrin 3
1.88	0.00701	CNTN1	Contactin 1
1.67	0.007549	SCEL	sciellin
1.91	0.007777	IL1RN	interleukin 1 receptor antagonist
1.51	0.007882	EPB41L3	erythrocyte membrane protein band 4.1-like 3
1.58	0.008332	CNTN1	contactin 1
1.50	0.00847	ENTPD4	ectonucleoside triphosphate diphosphohydrolase 4
1.50	0.009824	NFYA	nuclear transcription factor Y, alpha

1.68	0.00986	SCEL	sciellin
1.78	0.011054	CNTN1	Contactin 1
1.51	0.011171	BBOX1	butyrobetaine (gamma), 2-oxoglutarate dioxygenase 1
1.66	0.012022	KRT4	Keratin 4
1.52	0.012504	LOC202181	hypothetical protein LOC202181
1.84	0.013414	SPRR1B	small proline-rich protein 1B (cornifin)
2.46	0.0148	PTPRZ1	protein tyrosine phosphatase, receptor-type, Z polypeptide 1
1.68	0.015609	KRT4	keratin 4
1.52	0.016746	CCBL2	cysteine conjugate-beta lyase 2
1.76	0.019041	DST	dystonin
1.60	0.022237	VSNL1	visinin-like 1
1.95	0.022642	SPRR1A	small proline-rich protein 1A
1.67	0.023611	SERPINB3	serpin peptidase inhibitor, clade B (ovalbumin), member 3
2.03	0.025302	SPRR1A	small proline-rich protein 1A
1.58	0.025745	CLTA	clathrin, light chain (Lca)
1.79	0.027657	A2ML1	alpha-2-macroglobulin-like 1
2.31	0.028093	GJA1	gap junction protein, alpha 1, 43kDa
1.64	0.028587	S100A12	S100 calcium binding protein A12
1.71	0.028884	GPX2	glutathione peroxidase 2 (gastrointestinal)
1.84	0.029502	DSG3	desmoglein 3 (pemphigus vulgaris antigen)
1.54	0.029846	ZNF117	zinc finger protein 117
1.51	0.031188	OTUB2	OTU domain, ubiquitin aldehyde binding 2
1.55	0.032795	DST	dystonin
2.66	0.034061	C14orf34	chromosome 14 open reading frame 34
1.90	0.039329	SPRR3	small proline-rich protein 3
1.62	0.041107	MYEOV	myeloma overexpressed
1.56	0.042799	RASSF6	Ras association (RalGDS/AF-6) domain family member 6
1.59	0.047392	NCF2	neutrophil cytosolic factor 2
1.95	0.048364	SPRR3	small proline-rich protein 3

Supplementary Figure S1. Expression of ETV5 mRNA (**a**) and FGFR3 and ETV5 protein (**c**) in the FGFR3-mutant cancer cell line 97-7 after silencing (shFR3) and re-expression (shFR3+S249C) of mutant FGFR3, compared to control cells transduced with the empty vector, and cell expressing a scrambled shRNA (Scr). Expression of ETV5 mRNA (**b**) and FGFR3 and ETV5 protein (**d**) in the FGFR3-mutant cancer cell line UMUC14 after silencing (shFR3) of mutant FGFR3, compared to control cells transduced with the empty vector, and cell expressing a scrambled shRNA (Scr). ETV5 mRNA was relatively quantified using Taqman Real-Time RT-PCR with SDHA as internal control. ETV5 and FGFR3 proteins were visualized by Western Blotting with specific antibodies using beta-actin as loading control. All experiments were repeated in triplicate.

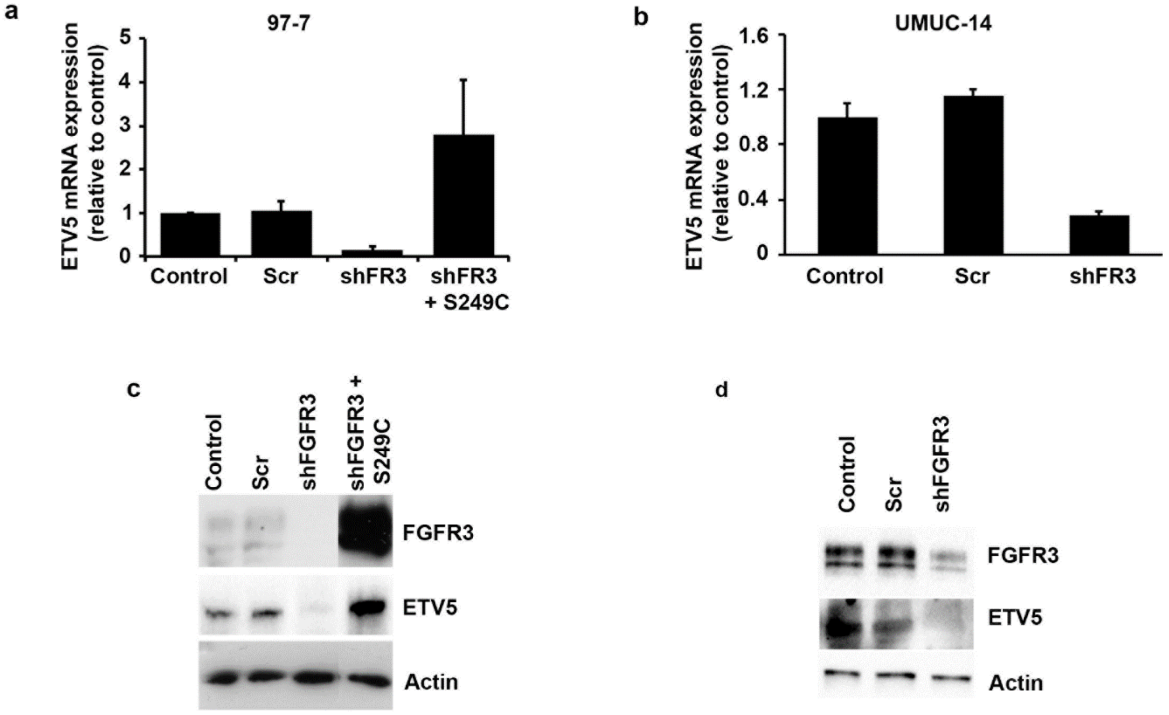

Supplementary Figure S2. Expression of ETV5 and known ETV5 target genes in 97-7, CAL29, UMUC14 and MGHU3 cells with ETV5 knockdown compared to control cells transduced with the empty vector. mRNA expression levels were relatively quantified using Taqman Real-Time RT-PCR with SDHA as internal control. Experiments were repeated in triplicates.

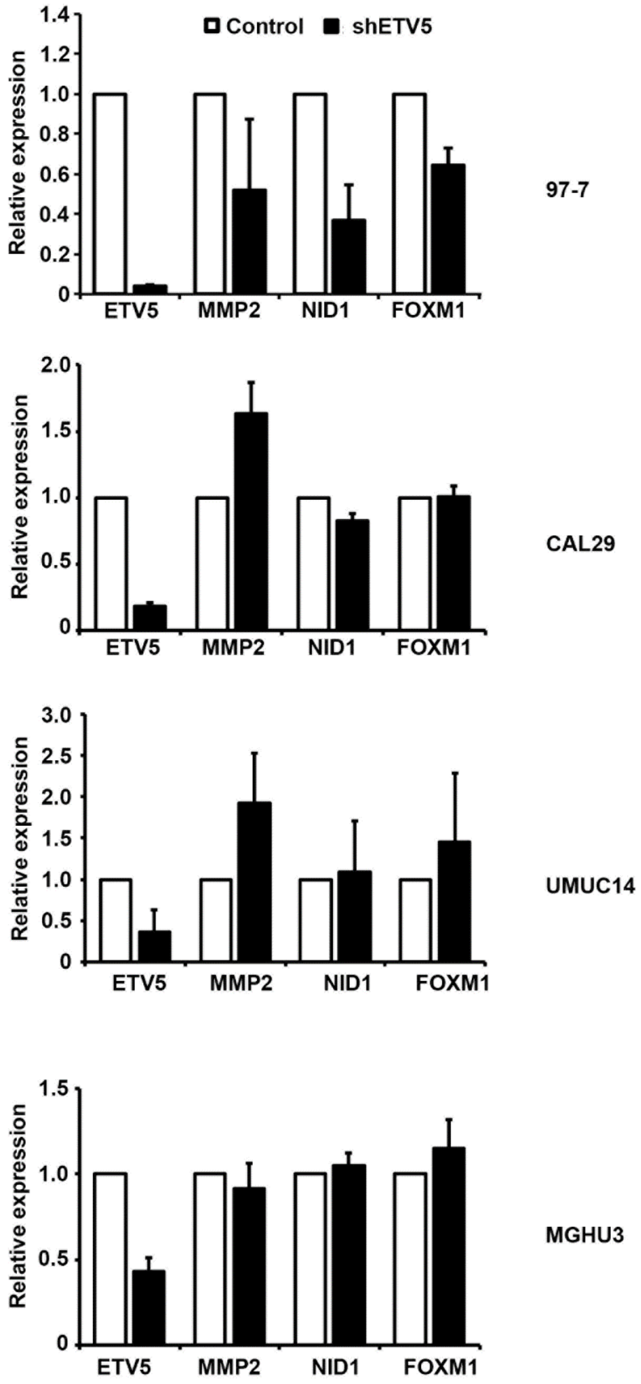

Supplementary Figure S3. Correlation between ETV5, TAZ, CYR61 or CTGF expression in bladder tumours. Data from two large publically available bladder tumour studies [47, 48] was interrogated for correlation between ETV5 and TAZ, CTGF and CYR61 mRNA expression levels, using R2 Genomics Analysis and Visualization Platform (http://r2.amc.nl). Gene expression levels are expressed as log 2 of the normalized intensity values.

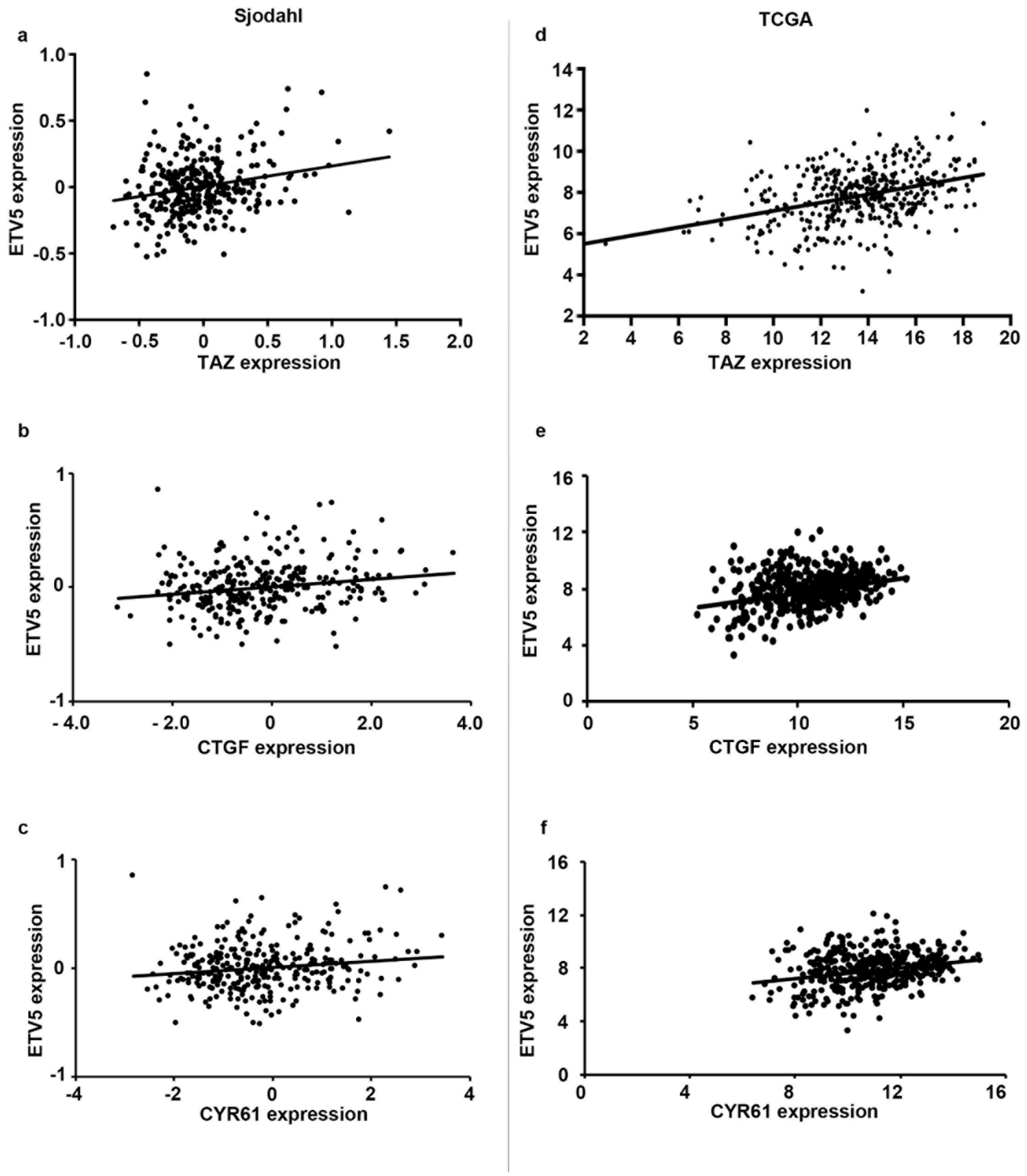

Supplementary Figure 4.

Images of all uncropped blots.

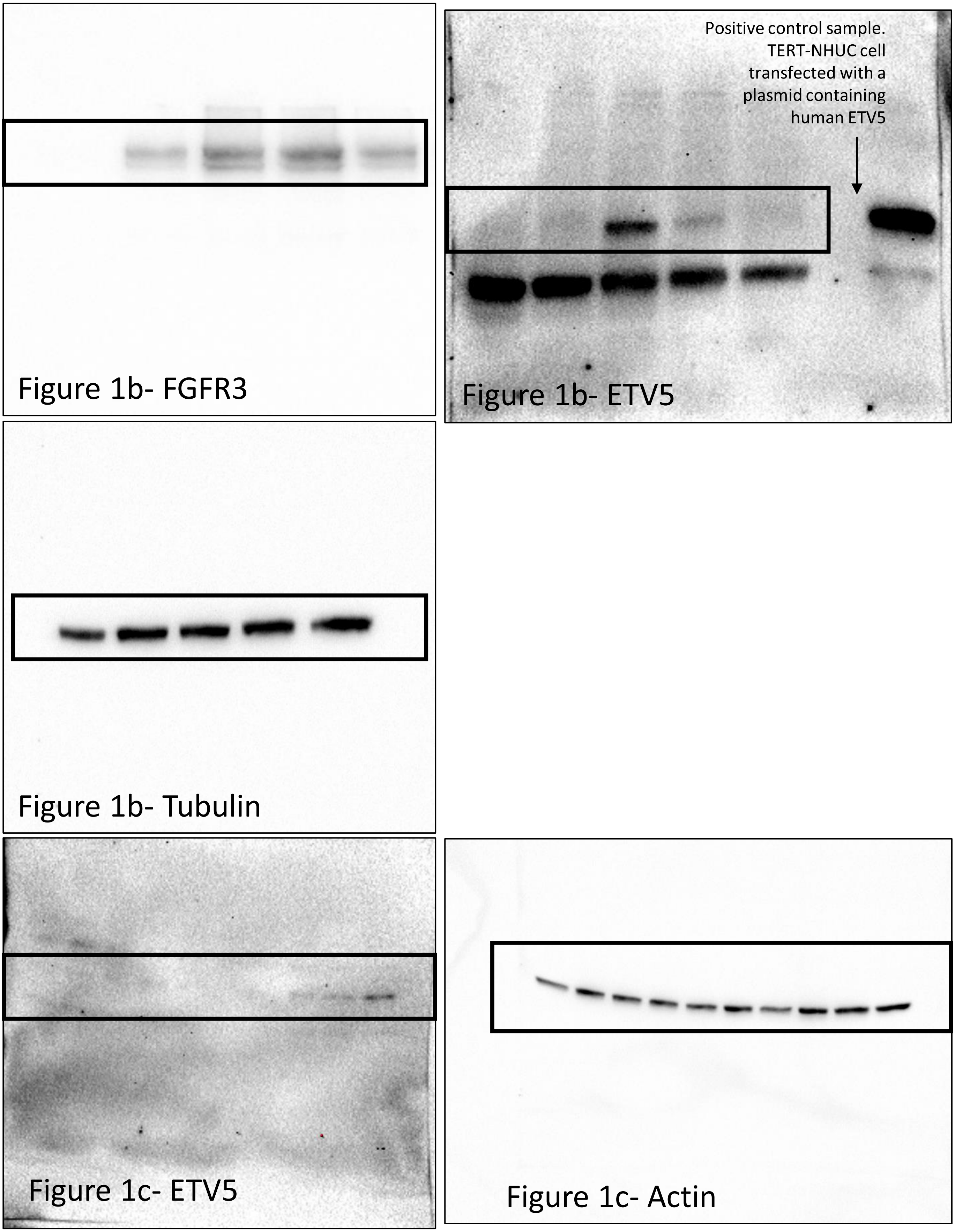

Figure 1 unadjusted original blots, with sections included in main figure highlighted by the boxes

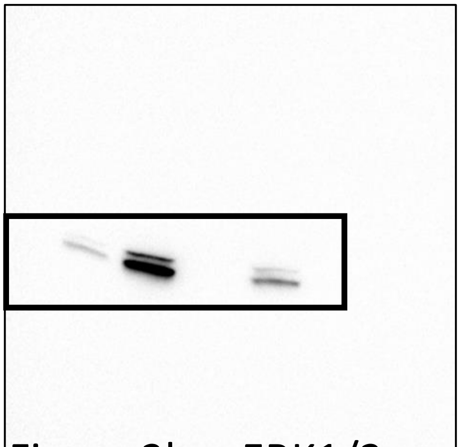

Figure 2b- pERK1/2

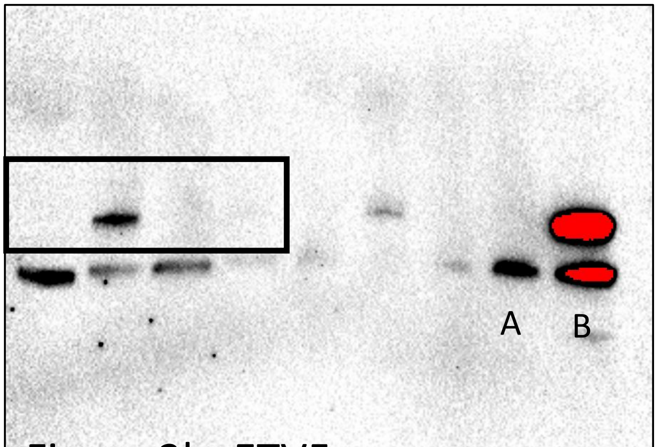

Figure 2b- ETV5

A. Negative control. TERT-NHUC transfected with an empty plasmid.
B. Positive control. TERT-NHUC cell transfected with a plasmid containing human ETV5

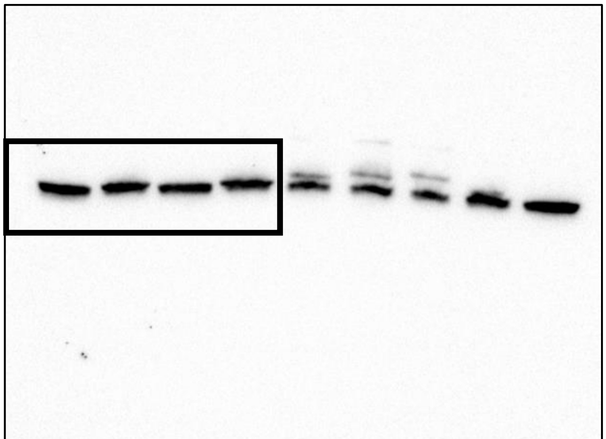

Figure 2b- Tubulin

Figure 2 unadjusted original blots, with the sections included in main figure highlighted by the boxes

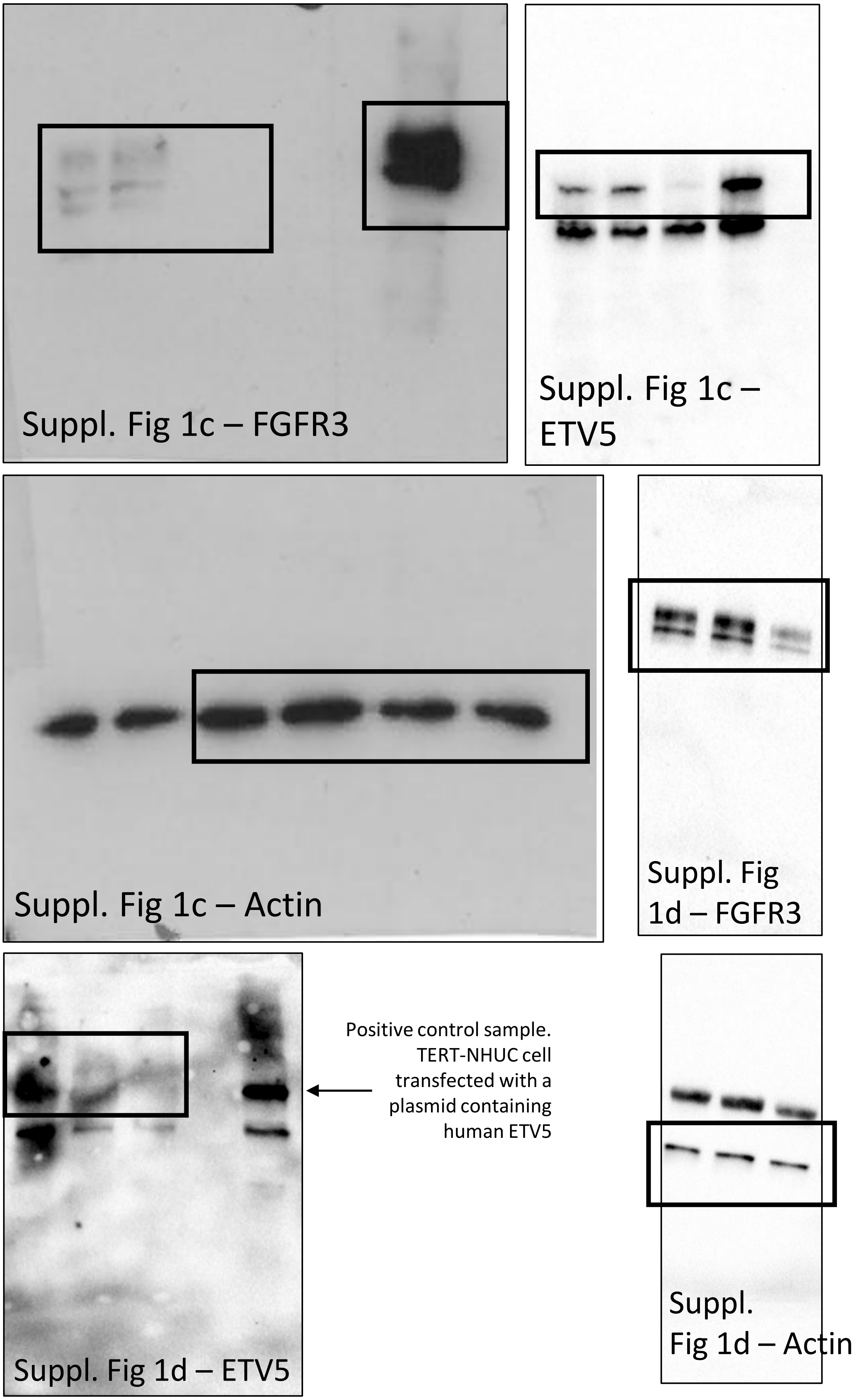

Suppl. Fig 1 unadjusted original blots, with the sections included in main figure highlighted by the boxes

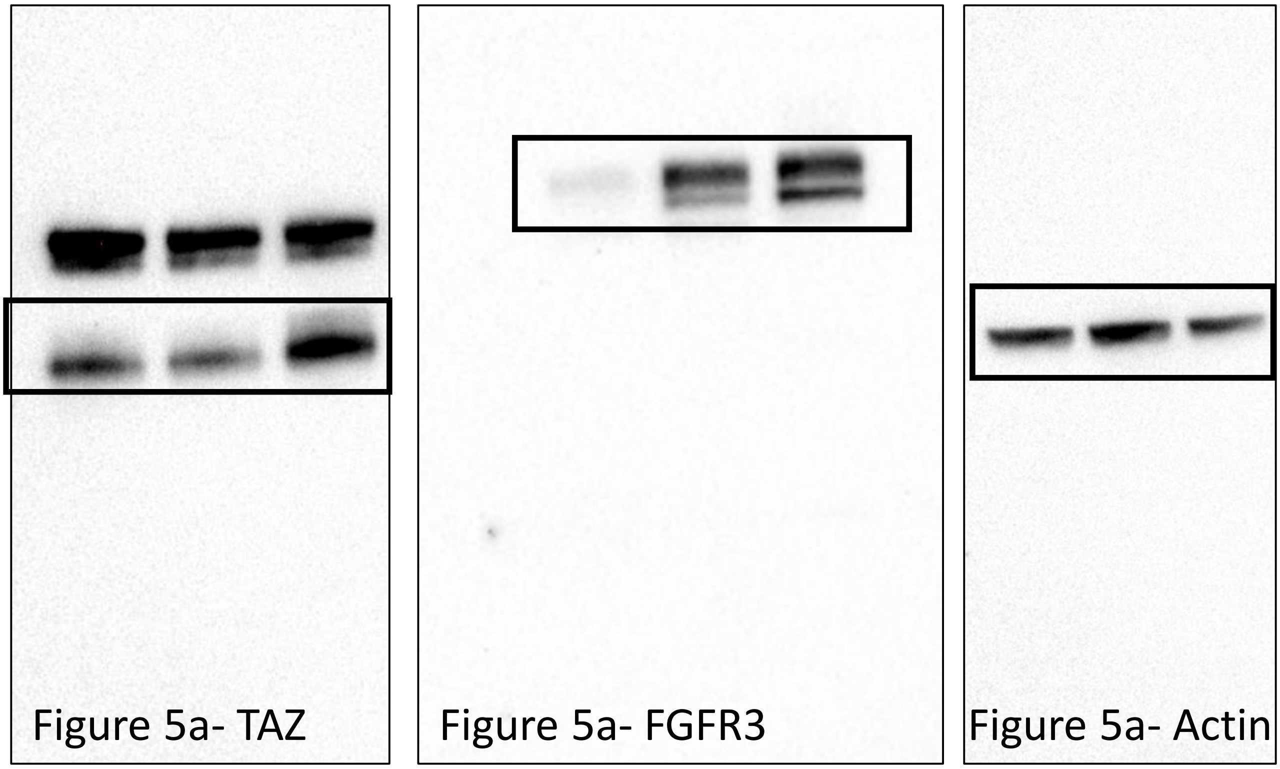

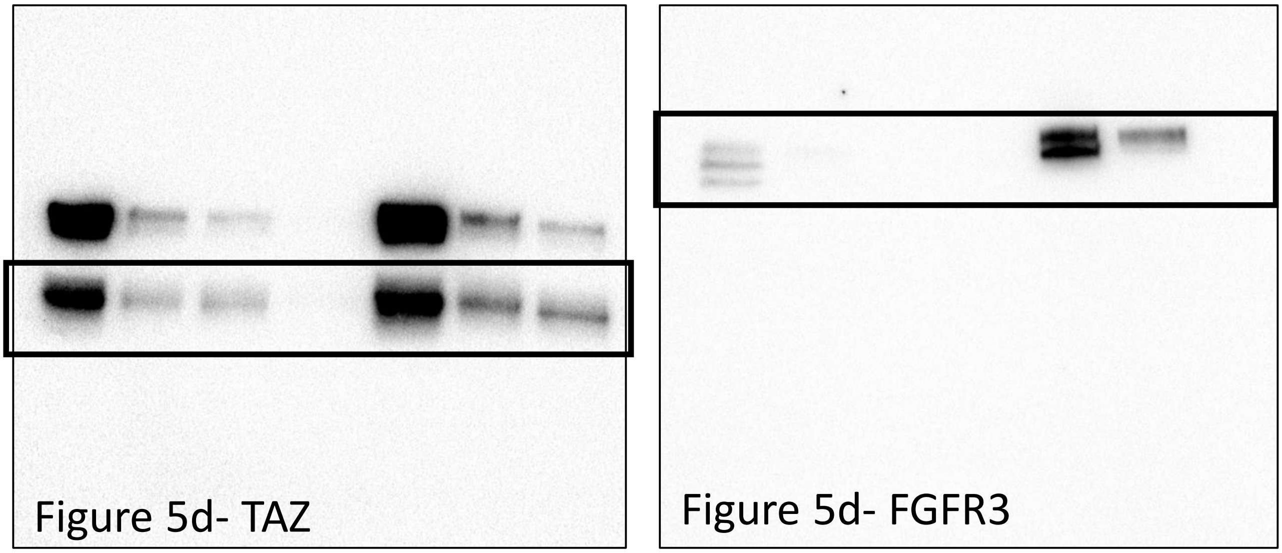

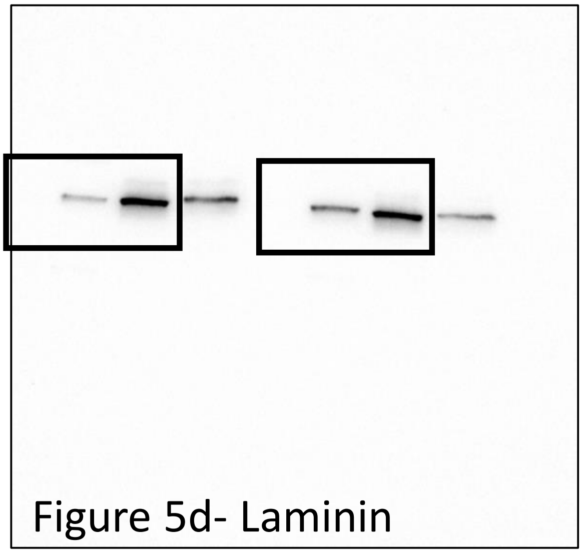

Figure 5 unadjusted original blots, with the sections included in main figure highlighted by the boxes

Note: the TAZ antibody recognizes both YAP (70 KDa) and TAZ (50KDa) as these are highly homologous protein of the same family. TAZ is the lower molecular weight band.
